# Impact of chronic allergic rhinitis on bite force and electromyographic activity of masseter and temporalis muscles of adult women

**DOI:** 10.4317/jced.56660

**Published:** 2020-05-01

**Authors:** Natalia-Augusta-Ferreira Bordignon, Simone Regalo, Paulo-Batista de Vasconcelos, Marcos-Vinicios-Ribeiro Prandi, Takami-Hirono Hotta, Ligia-Maria-Napolitano Gonçalves, Isabela-Hallak Regalo, Selma Siéssere, Marcelo Palinkas

**Affiliations:** 1 MS. Department of Basic and Oral Pathology, School of Dentistry of Ribeirão Preto, University of São Paulo, Brazil; 2 DDS, PhD, Professor. Department of Basic and Oral Pathology, School of Dentistry of Ribeirão Preto, University of São Paulo; National Institute and Technology - Translational Medicine (INCT.TM), São Paulo, Brazil; 3 DDS, Professor. Department of Dental Materials and Prosthodontic, Ribeirão Preto School of Dentistry, University of São Paulo, Brazil; 4 DDS, PhD, Professor. Department of Basic and Oral Pathology, School of Dentistry of Ribeirão Preto, University of São Paulo; Brazil; 5 DDS, PhD, Professor. Department of Basic and Oral Pathology, School of Dentistry of Ribeirão Preto, University of São Paulo; Faculty Anhanguera, Ribeirão Preto and National Institute and Technology - Translational Medicine (INCT.TM), São Paulo, Brazil

## Abstract

**Background:**

The aim of this study was to analyse the stomatognathic system of adult women with chronic allergic rhinitis by means of molar bite force and electromyographic activity of the masseter and temporalis muscles.

**Material and Methods:**

A total of 26 subjects were screened and divided into two distinct groups: chronic allergic rhinitis group (n = 13) and healthy control group (n = 13). Subjects were assessed by maximal molar bite force (right and left) and normalized electromyographic activity of mandibular tasks (rest, right and left laterality, protrusion and maximal voluntary contraction). Data were submitted to Student’s t test (*p*< .05).

**Results:**

There was significant difference in right (*p* = .03) and left (*p* = .04) maximal molar bite force with force reduction in the chronic allergic rhinitis group. There was significant difference in normalized electromyographic activity in maximal voluntary contraction in the right (*p* =.01) and left (*p* = .01) temporalis muscles, with increased electromyographic activity in the masticatory muscles for the chronic allergic rhinitis group.

**Conclusions:**

The results suggest that chronic allergic rhinitis in adult women promoted negative changes in the electromyographic activity of temporalis muscles in maximal voluntary contraction and maximal molar bite force.

** Key words:**Rhinitis, occlusal force, electromyography, masticatory muscles.

## Introduction

Inflammation is a biological process of organic response that occurs between organism and environment, often resulting from trauma, infections and episodes that threaten the human organismo (1,2). Allergic rhinitis is a chronic inflammatory dysfunction of the mucosa of nasal lining (3) mediated by immunoglobulin E, after presenting a reaction to exposure to allergens. The most characteristic symptoms are nasal congestion, anterior and posterior rhinorrhea, sneezing, nasal itching and hyposmia (4,5).

It is considered a public health problem that affects 10-25% of the world’s population (6). The male gender develops more severe and persistent forms of allergic rhinitis and the highest frequency is in the female gender (7). It has increased among the population over the years, although many people do not recognize it as a disease and thus do not seek adequate medical care, therefore being underdiagnosed or undertreated (8).

It is known that the human organism presents a complex interaction between the static and dynamic structures and the stomatognathic system, in turn, also demonstrates this complexity of functional interaction in order to keep breathing, chewing, swallowing, phonation and suction in Harmony (9,10).

Any functional alteration of orofacial dynamic structures, such as local inflammations, may compromise adjacent structures. Therefore, the aim of this study was to evaluate the maximal molar bite force and electromyographic activity of the masseter and temporalis muscles of adult women with chronic allergic rhinitis. The null hypothesis of this study is that chronic allergic rhinitis in adult women does not influence orofacial functional dynamics.

## Material and Methods

-Subject and study design

This research was approved by the Research Ethics Committee (process No. 02735812.9.0000.5419), based on Resolution 466/2012 of the Brazilian National Health Council. Informed consent was obtained from all subjects included in the study.

The a priori sample size was calculated considering the level of α = .05, a power of 96% for the main result of electromyographic activity (µV/s), during the total lung capacity maneuver of Steer *et al.* (11) (mean [standard deviation] parasternal intercostal muscles: healthy subjects group, 90.0 [28.3] and subjects with uncontrolled respiratory inflammation, 48.0 [32.5]). The effect size was 1.37. The minimum sample size obtained was 26 subjects (13 for each group). Sample size was calculated using the G* Power 3.1.9.2 software (Franz Faul, Kiel University, Kiel, Germany).

At the beginning of this study, 60 subjects, aged 18 to 40 years, with normal occlusion (Angle Class I). After the exclusion criteria have been applied, 13 subjects with a confirmed diagnosis of chronic allergic rhinitis by an otolaryngologist were selected.

The period of inflammation of the lining of the nasal lining was more than four days a week and lasted for more than four weeks (12). All subjects with chronic allergic rhinitis used vasodilator decongestants and were mouth breathing.

In the anamnesis, a clinical form was used to obtain information regarding personal data, medical and dental history, presence of systemic diseases and parafunctional habits. Research Diagnostic Criteria for Temporomandibular Disorders (RDC / TMD) was used to rule out temporomandibular disorders in the subjects (case and control).

Exclusion criteria for the chronic allergic rhinitis group were as follows: subjects with neurological and systemic disorders (n = 04), temporomandibular disorders (n = 07), presence of poorly fitting fixed dental prostheses with cavities 

that cause infiltrations (n = 03); presence of periodontal disease determined by simplified periodontal record (n = 06), absence of first permanent molars (n = 07); parafunctional habits (n = 08), mandibular tori which is associated with changes in the function of the stomatognathic system (13) (n = 01), use of muscle relaxants that could interfere with neuromuscular physiology (n = 07); respiratory diseases, with viral infection (cold and flu) in the last month (n = 02), bronchitis (n = 01) and asthma (n = 02).

To define the control group, healthy adult women had all teeth (except third molars), normal occlusion, Angle Class I, and no temporomandibular dysfunction (RDC / TMD). The control group was paired subject-to-subject by age and body mass index with the chronic allergic rhinitis group (Table 1).

**Table 1:**
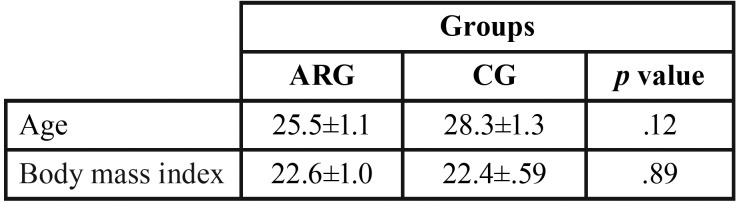
Comparison of means: age (years) and body mass index (Kg/m2) for chronic allergic rhinitis group (ARG) and control group (CG) using t test (*p*< .05).

The evaluation of electromyographic and bite force records were performed by a single trained professional. Intra-examiner calibration was performed for all analyzes of this study. The reliability of the intra-rater was good by calculating the intra-class coefficient (ICC). Reliability was considered acceptable for electromyographic activity (ICC = 0.936) and molar bite force (ICC = 0.928).

-Molar bite force analysis

The bite force was evaluated by measuring the maximal bite force, using the IDDK digital dynamometer (Kratos, Cotia, SP, Brazil) adapted to the buccal condition, which allowed measuring the maximal force applied in Newtons (14). The digital dynamometer was positioned in the region of the upper and lower first permanent molars (right and left side), because it develops greater force in the buccal cavity (15).

To record the measurements, the subjects remained seated in a comfortable office chair with their arms extended over their bodies and their hands resting on their thighs. Guidance and training to bite the dynamometer rods was ensured to promote greater credibility of the methodology. The dynamometer rods were protected by disposable latex finger straps (Wariper-Sp) and cleaned with alcohol for biosafety reasons (16).

Three maximal bites on each side (right and left) were performed with a 2-minute interval between each measurement. The bite force value was conFigured by the highest force value presented from the three measurements of each side of the dental arch (17).

-Analysis of electromyographic activity

Electromyographic activity was recorded to evaluate the activation pattern of the masseter and temporalis muscles using Trigno wireless electromyography equipment (Delsys Inc., Boston, MA, USA). The sensors were adjusted in the 20-450 Hz range and 80dB common mode rejection rate.

The sensors were positioned in the masseter and temporalis muscles by the same operator trained according to the SENIAM recommendations (Surface EMG for non-invasive assessment of muscles) (18). Specific maneuvers of maximal isometric contraction were performed to determine the best collection points for electromyographic signals. Before the positioning of the sensors, the skin went through an alcohol sanitization process to reduce the impedance, and the sensors were fixed after a few minutes of the procedure (19).

The Frankfurt plane was used as the head positioning parameter. Instructions and explanations were given, requesting calm and tranquility during the exam. During the tests, the subjects sat in a comfortable office chair, maintaining their upright posture, feet flat on the floor and arms resting on their legs.

The electromyographic recordings followed the following protocol of mandibular tasks: rest (4 s), protrusion (4 s), right laterality (4 s), left laterality (4 s), maximal voluntary contraction clenching (4 s) and maximal voluntary contraction clenching with inert material (4 s). The inert material consisted of paraffin sheet (Parafilm M, Pechiney Plastic Packaging, Batavia, IL, USA) inserted between the occlusal faces of the upper and lower first molars on the right and left sides of the dental arch (20).

The gross electromyographic signal (microvolts / second) was applied to derive electromyographic amplitude values obtained by calculating the square root mean used for mandibular tasks. Maximal voluntary contraction with Parafilm M was used for normalization of electromyographic data.

-Method Error

For the reliability of the results, Dahlberg’s formula was used to demonstrate casual error in this study. Measurements of molar bite force and normalized electromyographic activity were calculated using the recordings of five subjects and obtained during two different sessions with an interval of seven days. A small difference was observed in measurements between the first and second session on bite force with the average of the three bites calculated for right and left side (5.21%) and electromyographic activity (3.74%).

-Statistical analysis

In the analysis of the results, the data showed normal distribution (Shapiro–Wilk normality test: *p* ≤ .05). Data on maximal molar bite force and normalized electromyographic data were submitted to statistical analysis using IBM SPSS Statistics for Windows, version 22.0 (IBM SPSS, IBM Corp., Armonk, NY, USA). Results were obtained by descriptive analysis (mean and standard error) for each variable. Values were compared by using the Student’s t-test, with statistical significance set at *p*-values < .05.

## Results

Table 2 showed the mean of right and left maximal molar bite force between the groups. There was significant difference in right (*p* = .03) and left (*p* = .04) maximal molar bite force with force reduction in the chronic allergic rhinitis group.

**Table 2:**
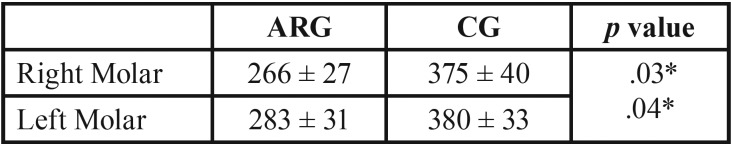
Means, standard errors (±) and statistical significance (*p*< .05*) of the right and left molar bite force (Newtons) for chronic allergic rhinitis group (ARG) and control group (GC).

Table 3 showed significant difference in normalized electromyographic activity in maximal isometric contraction in the right (*p* = .01) and left (*p* = .01) temporalis muscles with increased electromyographic activity in the masseter and temporalis muscles for the chronic allergic rhinitis group.

**Table 3:**
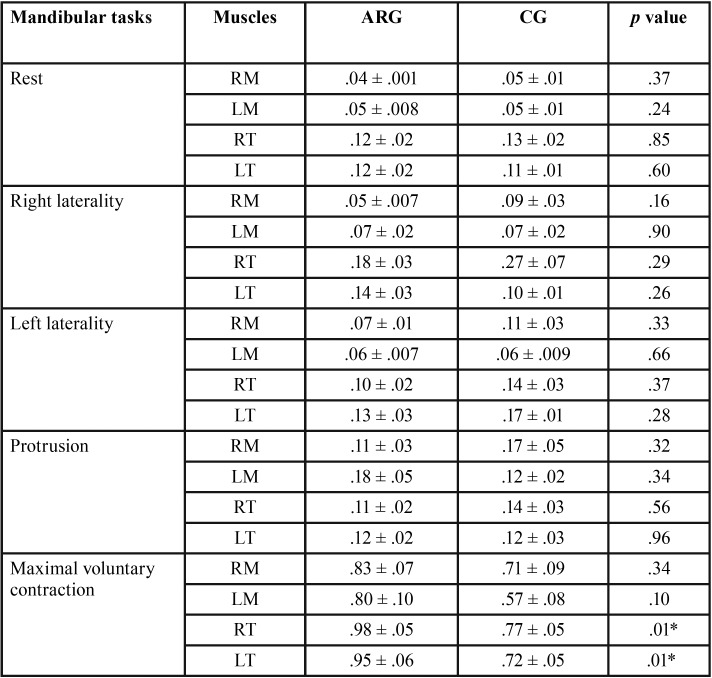
Means, standard errors (±) and statistical significance (*p*< .05*) of the normalized electromyographic data averages of the right masseter (RM), left masseter (LM), right temporal (RT) and left temporal (LT) for chronic allergic rhinitis group (ARG) and control group (CG) in the mandibular tasks.

## Discussion

The null hypothesis was partially rejected when it observed significant negative influence of chronic allergic rhinitis on maximal isometric contraction activity of temporalis muscles and maximal molar bite force.

In this study, it was observed that the electromyographic activities of the masseter and temporalis muscles were similar between groups during mandibular rest. This fact reveals the existence of postural maintenance with activation of muscle fibers in the healthy organism (21,22) and functional alteration (23).

During the protrusion, the chronic allergic rhinitis group demonstrated an adequate neuroanatomic pattern of muscle activation to maintain the postural position (21). In this condition, changes in normalized electromyographic means of the masseter muscles were observed between the groups. This situation may be related to the functional imbalance of the stomatognathic system due to the oral breathing pattern (24). The buccal breathing pattern in subjects affected by upper airway inflammation promotes muscle compensations that trigger functional imbalance (25).

During right and left laterality, the chronic allergic rhinitis group had lower normalized electromyographic means than the control group, without significant difference. The results presented are directed at the muscles that effectively participated in the neuroanatomic muscle activation pattern.

In this pattern there was greater electromyographic activation of the temporal muscle on the same side of the mandible that extends to the functional side, while in the masseter muscle the highest activation was contralateral to movement (23). The hypothesis for the lowest normalized electromyographic means of the masticatory muscles may be related to the buccal breathing pattern (26).

Normalized electromyographic activity in the present investigation was measured in the maximal isometric contraction of the masseter and temporalis muscles in both groups. Higher electromyographic means were demonstrated in the group with chronic allergic rhinitis when compared to the control group, with significant difference for the temporalis muscles.

Muscle performance may be influenced by topical nasal decongestants containing vasoconstrictors used to treat allergic rhinitis. Studies indicate that small doses of vasoconstrictors, for example adrenaline, constantly released into the bloodstream, promote acute vasodilation, increasing the caliber of vessels and arteries present in skeletal striated muscle through beta-adrenergic mechanism (27).

Chronic administration of substances that help treat respiratory diseases dilate blood vessels, causing hypertrophy and increased muscle activity in controlled isometric contractions, with slow to fast fiber transition (28). In this study, the group with chronic allergic rhinitis made continuous use of nasal decongestants with vasoconstrictors.

Considering the means of normalized electromyographic activity of the masticatory muscles of the group with chronic allergic rhinitis and control, it was observed that there was an increase of temporal muscle activity in relation to masseter muscles in mandibular tasks, except for protrusion, with no significant difference.

Regarding the functional role of the masseter and temporalis muscles, science proposes that the masseter muscle is more powerful and functional in this neuroanatomic movement than the temporal muscle, which has the primary utility of maintaining mandibular positioning (29).

Psychophysiological processes triggered by stressors and daily tensions are known to influence the tension of skeletal striated muscles, being able to inappropriately stimulate myoelectric activity (30), inducing changes in the components of the stomatognathic system in healthy subjects and those who report signs of functional changes (21).This fact could explain the increase in temporal muscle activity in relation to masseter muscle.

Mouth breathing-related inflammatory functional dysfunctions are reported to promote decreased force (31). Molar bite force is defined by the effect of the association between components of the stomatognathic system that are monitored by the central nervous system indicating their functional condition. The results of this study showed that adult subjects with chronic allergic rhinitis had lower right and left maximal molar bite force, with significant difference.

The present study had some limitations with the impossibility of controlling variables that could interfere with the results such as vasoconstrictor concentration in the bloodstream. As practical implications, we can report that knowledge of the negative impact of allergic rhinitis on the stomatognathic system alerts health professionals to be more cautious in rehabilitative clinical treatments, because the stomatognathic system is compromised and may further aggravate the patient’s clinical condition.

## Conclusions

The results suggest that chronic allergic rhinitis causes negative functional changes in the stomatognathic system, especially in the maximal isometric electromyographic activity of the masticatory muscles and in the maximal molar bite force.
